# Circular RNA *circ_0000517* Facilitates The Growth and Metastasis of Non-Small Cell Lung Cancer by Sponging *miR-326/miR-330-5p*

**DOI:** 10.22074/cellj.2021.7913

**Published:** 2021-10-30

**Authors:** Qiyan Tan, Changyu Liu, Ying Shen, Tao Huang

**Affiliations:** Department of Laboratory, Hainan People’s Hospital, Haikou, Hainan, China

**Keywords:** miR-326, miR-330-5p, MMP2, Non-Small Cell Lung Cancer

## Abstract

**Objective:**

There is growing evidence showing that circular RNAs (circRNAs) are crucial regulators in modulating the biological behavior of tumors. This work is aimed to probe the role of circ_0000517 in non-small cell lung cancer (NSCLC) and to elucidate its mechanism of action.

**Materials and Methods:**

In this experimental study, the differentially expressed circRNAs in NSCLC were screened using the GEO database (GSE158695). *Circ_0000517, miR-326, miR-330-5p,* and *MMP2* expression levels were determined by quantitative real-time polymerase chain reaction (qRT-PCR) analysis and Western blot. The proliferation, apoptosis, migration, and invasion of NSCLC cells were detected by CCK-8, flow cytometry, and transwell assays. RNA immunoprecipitation (RIP), RNA pull-down, and dual-luciferase reporter gene assays were performed to clarify the association between the *circ_0000517* and *miR-326/miR-330-5p*.

**Results:**

* Circ_0000517* was shown to be up-regulated in NSCLC tissues and cell lines. The up-regulation of *circ_0000517* is closely associated with advanced clinical stage of cancer, lymph node metastasis, and poor prognosis in NSCLC patients. *Circ_0000517* knockdown impeded the proliferation, migration, and invasion of NSCLC cells and enhanced their apoptosis. Mechanistically, circ_0000517 was demonstrated to up-regulate MMP2 expression via decoying *miR-326* and *miR-330-5p* to facilitate the malignant biological behaviors of NSCLC cells.

**Conclusion:**

This work reveals that *circ_0000517* is implicated in NSCLC cell growth and metastasis through the modulation of miR-326/miR-330-5p/MMP2, providing novel insights into the role of circRNAs in NSCLC progression.

## Introduction

Lung cancer (LC) is the most common type of tumor and the chief cause of cancer-related death, worldwide (1). Non-small cell lung cancer (NSCLC) is the major subtype of LC, taking up more than 85% of all cases (2). Despite the progress in NSCLC therapy, the survival and prognosis of NSCLC patients are still unfavorable (3- 5). Hence, it is important to investigate the molecular mechanisms of the carcinogenesis and development of NSCLC.

CircRNAs are endogenous non-coding RNAs (ncRNAs) that form closed loops by covalently linking together the 3’ and 5’ ends of one or more exons (6). CircRNAs were discovered in RNA viruses as early as 1976 (7, 8). At first, they were thought to be the products of splicing errors (7). CircRNAs in mammals are with relative stability and high tissue- and cell-specific expression, exerting an important role in regulating the biological processes and pathogenesis (9, 10). For instance, *circ_001783* is overexpressed in breast cancer (BC) tissues and is remarkably linked to a heavier tumor burden and poorer prognosis in BC patients (11). Reportedly, knocking down circ_0000799 inhibits the proliferation and migration of bladder cancer cells* in vitro* and impedes tumor growth *in vivo* (12). *Circ_0000517* is a newly discovered circRNA that is abnormally overexpressed in hepatocellular carcinoma (HCC), and its expression is linked to adverse clinical outcomes (13). Nonetheless, the expression features of *circ_0000517*, its biological functions and its underlying mechanism in NSCLC are still unclear.

Competitive endogenous RNAs (ceRNAs) are RNA transcripts involved in "target mimetic" processes, also known as miRNA "sponges" or miRNA "decoys"(14). It binds competitively to miRNAs through base complementarity with miRNA response elements, thereby decreasing the number of miRNAs targeting mRNAs (15). CircRNAs can function as effective miRNA sponges that disrupt mRNA translation and play a role in cancer progression (16, 17). For instance, *circ_0008039* enhances the proliferation and cycle progression of BC cells through regulating miR-432-5p/E2F3 axis (18). *Circ_0091570* up-regulates ISM1 expression as a sponge for miR-1307 to modulate HCC growth and metastasis (19). Nonetheless, it remains to be further investigated whether *circ_0000517* may also participate in the ceRNA network in NSCLC. 

In this study, the GSE158695 query from the GEO database is analyzed, and *circ_0000517* is discovered to be abnormally overexpressed in NSCLC. Moreover, the research reveals that knocking down *circ_0000517* impedes the proliferation, migration, and invasion of NSCLC cells and enhances apoptosis. Furthermore, we demonstrate that, *circ_0000517* works as a molecular sponge for *miR-326/miR-330-5p* to accelerate NSCLC progression.

## Materials and Methods

### Tissue specimens

In this experimental study, a total of 37 samples of NSCLC tissues and paired paracancerous non-tumor tissues were available from subjects who underwent surgical resection at Hainan People’s Hospital. The ages of the participants were from 36 to 65 years, and consent forms were signed by the patients. Before their surgeries, none of the participants had a history of other tumors or underwent radio/chemotherapy. This work was endorsed by the Ethics Committee of Hainan People’s Hospital (2019A-3C011). The tissue specimens were frozen in liquid nitrogen shortly after resection and preserved at -196˚C until being used.

### Cell lines

Human normal bronchial epithelial cell line (BEAS-2B) and NSCLC cell lines (H1650, H1299, A549, H1975, and HCC827) were obtained from the Shanghai Cell Bank of Chinese Academy of Sciences (Shanghai, China). BEAS-2B cell line and NSCLC cells were maintained in RPMI1640 (Gibco, Grand Island, NY, USA) supplemented with 10% fetal bovine serum (FBS, Gibco, Grand Island, NY, USA), 100 μg/ml streptomycin, and 100 U/ml penicillin (Invitrogen, Carlsbad, CA, USA) at 37˚C with 5% CO_2_ .

### Bioinformatics analysis

Gene expression data of GSE158695 were obtained from the NCBI GEO database and analyzed using the online software GEO2R to screen for differentially expressed circRNAs. GSE158695 contained 6 human samples, including 3 cases of NSCLC tissues and 3 cases of paracancerous tissues. Sangerbox software (Mugu Biotech Company, Hangzhou, China) was used for cluster analysis. The target miRNAs of circ_0000517 were projected by CircInteractome database and StarBase database.

### Cell transfection

The NSCLC cells were plated in a 6-well plate at 3×10^5^ cells/well. The cells were transfected with small interfering RNAs (siRNAs) targeting *circ_0000517* (si-circ_0000517#1/2/3) and the negative control siRNA (si-NC), miR-326/miR-330-5p mimic (miR-326/miR-330- 5p) and control mimic (miR-NC), miR-326/miR-330-5p inhibitor (miR-326 in /miR-330-5p in) and inhibitor NC (miR-NC in), which were synthesized by GeneCopoeia (Shanghai, China). Cell transfection was executed using Lipofectamine 2000 (Invitrogen, Carlsbad, CA, USA).

### Preparation of RNA and quantitative real-time polymerase chain reaction analysis 

Total RNA was extracted from cells and tissues using the TRIzol reagent (Invitrogen, Carlsbad, CA, USA). The subcellular fractions of NSCLC cells were separated using the PARIS Kit (Ambion, Austin, TX, USA). RevertAid™ First Strand DNA Synthesis Kit (Thermo Fisher Scientific, Waltham, MA, USA) was used for reverse transcription, and quantitative real-time polymerase chain reaction (qRT-PCR) was executed using an SYBR Green PCR Kit (Toyobo, Osaka, Japan) on the 7500 Fast Dx Real-Time PCR Instrument (Applied Biosystems, Foster City, CA, USA). *β-actin* was considered as a control for normalization. MicroRNA detection was conducted using a miDETECT A Track Kit (RiboBio, Guangzhou, China). The small nuclear RNA *U6* expression was employed as a control for normalization. The primers for this research were designed using Primer Premier 5 software. The sequences are presented in Table S1 (See Supplementary Online Information at www.celljournal.org).

### RNase R trypsinization experiment

In this study, 20 μg total RNA was incubated with or without RNase R (20 mg/mL, Epicentre Biotechnologies, Shanghai, China) for 15 minutes at 37˚C. Following that, qRT-PCR was implemented to determine circ_0000517 and linear *RPPH1* mRNA expressions.

### Cell proliferation experiment

Cell proliferation was examined using the Cell Counting Kit-8 (CCK-8, Dojindo, Tokyo, Japan) assay. Cells were plated in a 96-well plate (3×10^3^ cells/well), and cultured for 1, 2, 3 or 4 days. Next, 10 μL of CCK-8 solution was added to each well. Then the cells remained in culture for 1 hour. Next, the absorbance (OD) at 450 nm was determined using a microplate reader (Model 550, Bio-Rad Laboratories, Inc., Hercules, CA, USA).

### Flow cytometry analysis

Briefly, to analyze the cell cycle of the transfected cells, the cells were fixed using 75% ethanol and then dyed using propidium iodide (PI, BD Biosciences, San Diego, CA, USA). Then a flow cytometer (BD Biosciences, Franklin Lake, NJ, USA) was used to detect the cell cycle distribution. To analyze the apoptotic rate of transfected cells, a Annexin V-FITC/PI apoptosis detection kit (BD Biosciences, San Jose, CA, USA) was used. The cells were resuspended in 1× binding buffer, and then dyed with AnnexinV-FITC staining solution and PI staining solution in the dark for 15 minutes. Subsequently, the apoptotic cells were analyzed with the flow cytometer.

### Transwell experiment

In the Transwell experiment, approximately 1×10^4^ transfected cells were suspended in 200 μL of serum-free medium and positioned in the top compartment of each Transwell (8 μm pore size, Corning, NY, USA). Matrigel (BD Biosciences, San Jose, CA, USA) was used to cover the filter in invasion assay, but it was not used in the migration assay. The lower compartment was filled with the medium-containing 10 % FBS as the chemoattractant. The cells were cultured for 48 hours for the invasion experiment and 24 hours for the migration assay. Following that, the cells in the upper compartment were swabbed with cotton swabs, while those on the lower surface of the filter were fixed with 0.1% crystal violet. In three random areas, the number of cells on the filter was recorded under a light microscope (Olympus Corporation, Tokyo, Japan).

### Dual-luciferase reporter gene experiment

By inserting the wild-type or mutant-type sequence of *circ_0000517* or *MMP2 3ˊUTR* containing miR-326/ miR-330-5p complementary sites into the psiCHECK-2 vector (Promega, Madison, WI, USA), respectively, wild-type luciferase reporter plasmids (circ_0000517-WT and MMP2 3ˊUTR-WT) and their mutants (circ_0000517- MUT and MMP2 3ˊUTR-MUT) were generated. The luciferase reporter plasmids were co-transfected into 293T cells and plated in a 96-well plate with miR-326/ miR-330-5p mimics. The miR-NC was employed as the negative control. After 48 hours, the cells were collected and the Dual-Luciferase Assay System (Promega, Madison, WI, USA) was used to assess the activities of Firefly and Renilla luciferase. The relative luciferase activity was normalized to Renilla luciferase activity.

### RNA immunoprecipitation assay

The RNA immunoprecipitation (RIP) experiment was executed with an EZ-Magna RIP Kit (Millipore, Billerica, MA, USA). A549 and H1299 cells were lysed in RIP lysis buffer plus cocktail (Roche Diagnostics, Shanghai, China). Supernatants were then incubated with protein A/G magnetic beads coupled with anti-Ago2 or IgG antibodies (Millipore, Billerica, MA, USA). After the immunoprecipitate was incubated with Proteinase K, qRT-PCR was performed to analyze the enrichment of miR-326 and miR-330-5p.

### RNA pull-down experiment

By using Biotin RNA Marking Mix (Roche), RNAs were biotin-labeled. After that, the biotinylated RNAs were incubated with A549 and H1299 cell lysates, followed by the incubation of M-280 streptavidin magnetic beads (Invitrogen, San Diego, CA, USA). After rinsing with RNase-free lysis buffer, the RNAs were extracted according to the manufacturer’s instructions, and the enrichment of *circ_0000517* was evaluated by qRT-PCR.

### Western blot

Total cellular protein was isolated using RIPA lysis buffer and stored on ice after the cells were washed with cold phosphate buffer saline (PBS, Sigma-Aldrich, Louis, MO, USA). Twenty µg of proteins per group were separated with 10% sodium dodecyl sulfate polyacrylamide gel electrophoresis (SDS-PAGE) and transferred onto a polyvinylidene difluoride (PVDF) membrane (Millipore, Burlington, MA, USA) using semi-dry transfer method (Bio-Rad, Hercules, CA, USA). After the membranes were blocked with 5% defatted milk, the membranes were incubated with primary and secondary antibodies according to standard protocols. After that, the protein bands were visualized using the ECL detection kit (Tanon, Shanghai, China). The protein bands were normalized with β-actin. The primary antibodies used in this study were as follows: anti-matrix metalloproteinase-2 (MMP2) (Abcam, ab92536, 1:1000), and anti-β-actin (Abcam, ab7817, 1:3000).

### Statistical analysis

All of tests were executed in triplicate. All data were analyzed using SPSS version 19.0 software (SPSS, Inc, Chicago, IL, USA). Student’s t test and one-way ANOVA were used to analyze the difference between two groups and among multiple groups, respectively. Correlations were measured by Pearson’s correlation analysis. Chi-square test was perform to analyze the association between clinical characteristics and *circ_0000517* expression levels. Kaplan-Meier survival curve was used to compare the prognosis of the NSCLC patients. P<0.05 signified statistical significance. 

## Results

### Circ_0000517 was up-regulated in NSCLC tissues and cell lines

**Fig.1:**
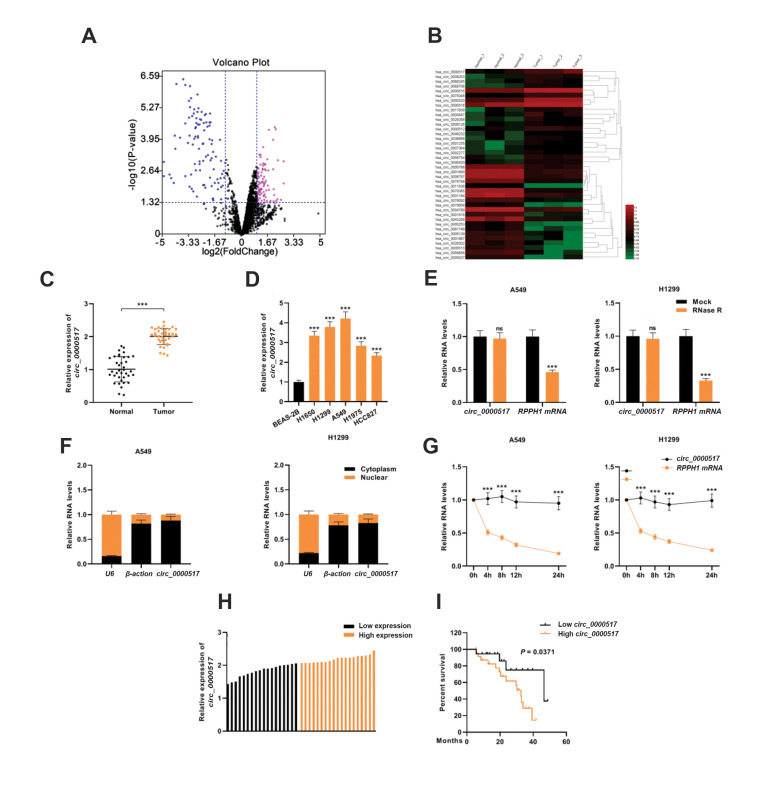
*Circ_0000517* was up-regulated in NSCLC. **A.** Differentially expressed circRNAs in NSCLC tissues (GSE158695) were presented in a volcano plot. The screening condition was ∣log2FC∣ >1 and P<0.05. **B.** Cluster heatmap of top 20 up- and down-regulated differentially expressed circRNAs (GSE158695). **C.** *Circ_0000517* expression level in 37 paired NSCLC tissues and matched adjacent normal tissues were examined by qRT-PCR. **D.** *Circ_0000517* expression in diverse human NSCLC cell lines (H1650, H1299, A549, H1975 and HCC827) and the human normal bronchial epithelial cell line (BEAS-2B) was examined by qRT-PCR. **E.** The relative RNA levels were examined by RT-qPCR after treatment with RNase R- or RNase R+ in total RNAs. **F. ***Circ_0000517* expression in the nuclei and cytoplasm of A549 and H1299 cell was examined by qRT-PCR. Our two controls were *β-actin*, which was mostly localized in the cytoplasm, and U6, which was mainly localized in the nuclei. **G.** qRT-PCR was executed to detect the relative levels of *circ_0000517* and *RPPH1* mRNAs at each time point after actinomycin D treatment. **H. **The 37 NSCLC patients were divided into low (n=18) and high expression groups (n=19) according to the median level of *circ_0000517* as the cut-off value. **I. **Kaplan-Meier survival curve was used to analyze the overall survival time of NSCLC patients (in TCGA database) with high or low *circ_0000517* expression levels. ***; P<0.001, NSCLC; Non-small cell lung cancer, and qRT-PCR; Quantitative real-time polymerase chain reaction.

At first, a GEO microarray dataset (GSE158695) was analyzed to find candidate genes in three pairs of human NSCLC tissues and normal lung tissues. The result suggested that the expression levels of 84 circRNAs were up-regulated and the expression levels of 101 circRNA expression were down-regulated in NSCLC tissues (P<0.05,∣Log2(Change fold)∣>1, Fig.1A). The top 20 up- and down-regulated circRNAs were exhibited in the heat map (Fig .1B), where *circ_0000517* was remarkably up-regulated in NSCLC tissues (Log2FC=2.665316, P<0.001). Additionally, *circ_0000517* expression in 37 pairs of NSCLC tissues and non-tumor tissues was detected by qRT-PCR analysis. The results showed that *circ_0000517* expression was higher in NSCLC tissues than in paracancerous normal tissues (Fig .1C, P<0.001). Moreover, relative to the normal bronchial epithelial cell line (BEAS-2B cells), *circ_0000517* expression in NSCLC cell lines (H1650, H1299, A549, H1975, and HCC827 cells) was markedly up-regulated (Fig .1D, P<0.001). Additionally, we showed that *RPPH1* was significantly degraded after RNase R treatment, but RNase R could not degrade *circ_0000517*, suggesting that *circ_0000517* had a closed-loop structure (Fig .1E). Furthermore, *circ_0000517* was found to be predominantly located in the cytoplasm of NSCLC cells (Fig .1F). Next, the half-life time of *circ_0000517* and *RPPH1* mRNA were measured in NSCLC cells treated with actinomycin D, which was used to restrain the transcription process. Our data showed that *circ_0000517* was more stable than *RPPH1* mRNA (Fig .1G). With the median expression level of *circ_0000517* as the cutoff value, the 37 NSCLC patients were divided into low and high expression groups (Fig .1H). A strong association was observed between *circ_0000517* expression and higher clinical stage, and lymphatic metastasis of the patients (Table 1). On the other hand, lower *circ_0000517* expression level in NSCLC tissues predicted a longer survival time of the patients (Fig .1I). 

**Table 1:** The relationship between *circ_0000517* and clinical characteristics in 37 NSCLC patients


Pathological indicators		Number of patients	Relative expression of circ_0000517		P value
			High expression	Low expression	

Gender					0.072
	Female	17	6	11	
	Male	20	13	7	
Age (Y)					0.254
	<47	17	7	10	
	≥47	20	12	8	
Histology					0.585
	Squamous cell carcinoma	32	17	15	
	Adenocarcinoma	5	2	3	
Clinical stage					0.013^*^
	I~II	19	6	13	
	II~III	18	13	5	
Tumor size (cm)					0.138
	<5	14	5	9	
	≥5	23	14	9	
Lymphatic metastasis					0.033^*^
	Yes	21	14	7	
	No	16	5	11	
Differentiation					0.124
	Well+moderate	22	9	13	
	Poor	15	10	5	


NSCLC; Non-small cell lung cancer and *; P<0.05.

### Knockdown of *circ_0000517* impeded NSCLC cell growth, migration, invasion, but enhanced apoptosis

**Fig.2:**
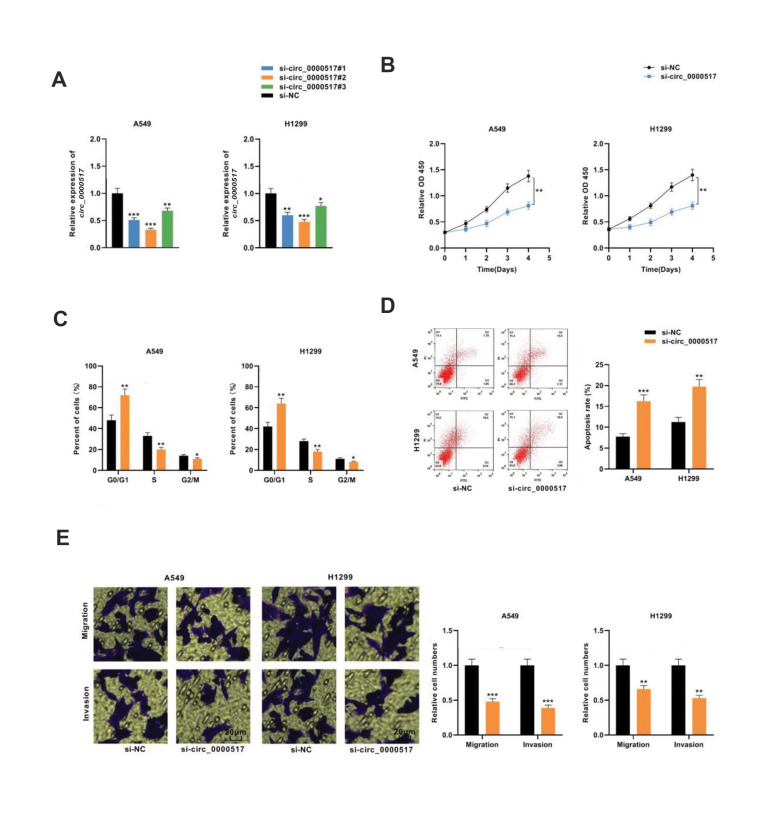
Knockdown of *circ_0000517* impeded the proliferation, migration, and invasion of NSCLC cells. **A.** Three siRNAs against circ_0000517 (si-circ_0000517#1, si-circ_0000517#2, and si-circ_0000517#3) were transfected into A549 and H1299 cells, and *circ_0000517* expression was detected by qRT-PCR. **B. **CCK-8 assay was employed to detect the proliferation of A549 and H1299 cells transfected with si-circ_0000517. **C.** Flow cytometry was used to detect the cell cycle distribution of A549 and H1299 cells transfected with si-circ_0000517. **D.** Flow cytometry was performed to detect the apoptosis rate of A549 and H1299 cells transfected with si-circ_0000517. **E. **Transwell experiment was done to detect the migration and invasion of A549 and H1299 cells transfected with si-circ_0000517 (scale bar: 20 µm). *; P<0.05, **; P<0.01, ***; P<0.001, NSCLC; Non-small cell lung cancer, and qRT-PCR; Quantitative real-time polymerase chain reaction.

As shown in Figure 1, *circ_0000517* expression was relatively high in A549 and H1299 cells among all tested NSCLC cell lines, and therefore these two cell lines were selected for the following functional assays. The *circ_0000517* knockdown cell models were generated by transfecting three siRNAs (si-circ 0000517#1/2/3) into A549 and H1299 cells (Fig .2A). Because the knockdown efficiency of si-circ_0000517#2 is the most significant, si-circ_0000517#2 was selected. CCK-8 experiment confirmed that *circ_0000517* knockdown significantly inhibited NSCLC cell proliferation compared with the control group (Fig .2B). Flow cytometry analysis revealed that knocking down *circ_0000517* increased the proportion of A549 and H1299 cells arrested in G0/G1 phase (Fig .2C). Additionally, knocking down *circ_0000517* remarkably increased the apoptotic rate of both cells relative to the control groups (Fig .2D). Moreover, the data of the Transwell experiments showed that knocking down circ_0000517 markedly reduced cell migration and invasion relative to the control (Fig .2E). Our findings indicated that knocking down *circ_0000517* impeded the proliferation, migration, and invasion of NSCLC cells, while enhancing apoptosis.

### *Circ_0000517* sponged *miR-326/miR-330-5p *in NSCLC cells

**Fig.3:**
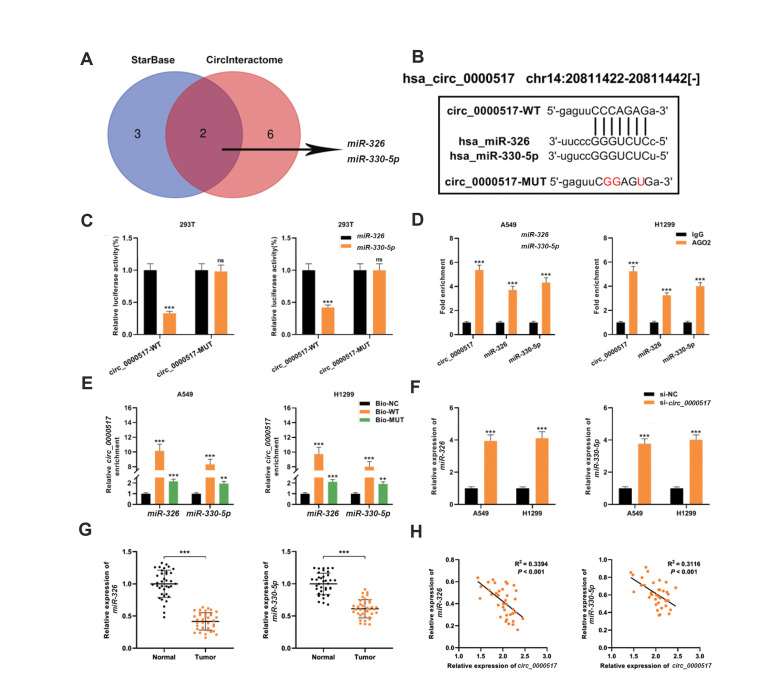
*miR-326 *and *miR-330-5p* were downstream targets of *circ_0000517*. **A.** Bioinformatics analysis predicted that the sequence of *miR-326* and *miR-330- 5p* matched the sequence of *circ_0000517*. **B. **The schematic diagram shows the putative *miR-326* and *miR-330-5p* binding sites with the circ_0000517, and the circ_0000517-WT and circ_0000517-MUT luciferase reporters that were constructed. **C. **Dual-luciferase reporter assays indicated that *miR-326* and *miR-330-5p* were the direct targets of circ_0000517. **D.** The complex containing circ_0000517 and *miR-326/miR-330-5p* in A549 and H1299 cells were immunoprecipitated by anti-Ago2 antibody in RIP assay. **E.** RNA pull-down experiment was carried out to verify the interactions between circ_0000517 and *miR-326/miR-330-5p*. **F. ***miR-326* and *miR-330-5p* expression levels in A549 and H1299 cells transfected with si-circ_0000517 were detected by qRT-PCR. **G. **qRT-PCR was employed to examine *miR-326* and *miR-330-5p* expression levels in 37 paired NSCLC tissues and matched adjacent normal tissues. **H. **Pearson’s correlation analysis was utilized to evaluate the correlations between *circ_0000517* expression and *miR-326/miR-330-5p* expression in NSCLC tissues. **; P<0.01, ***; P<0.001, ns; Not significant, NSCLC; Non-small cell lung cancer, and qRT-PCR; Quantitative real-time polymerase chain reaction.

The online prediction tools CircInteractome and StarBase were utilized to search for the downstream miRNAs that could bind to *circ_0000517*, and as a result, *miR-326* and *miR-330-5p* were predicted (Fig .3A). To prove the targeting relationship between *circ_0000517* and *miR-326/miR-330-5p*, wild-type circ_0000517 (circ_0000517-WT) and mutant circ_0000517 (circ_0000517-MUT) luciferase reporter vectors containing miR-326/miR-330-5p binding sites were constructed. (Fig .3B). MiR-326/miR-330-5p mimics substantially weakened the luciferase activity of circ_0000517-WT reporter, but had no effect on the luciferase activity of *circ_0000517*-MUT reporter (Fig .3C). RIP experiment suggested that *circ_0000517* and *miR-326/miR-330-5p *were enriched in microribonucleoprotein complexes containing Ago2 in A549 and H1299 cells (Fig .3D). Moreover, in both A549 and H1299 cells, *circ_0000517* could be pulled down by Bio-miR-326/miR-330-5p-WT, but not Bio-miR-326/miR-330-5p-MUT or Bio-NC (Fig .3E). In A549 and H1299 cells, knocking down *circ_0000517* markedly augmented *miR-326/miR-330-5p* expression (Fig .3F). Furthermore, *miR-326/miR-330-5p* was found to be substantially under-expressed in NSCLC tissues (Fig .3G); and was negatively correlated by *circ_0000517* expression (Fig .3H). Hence, we concluded that *circ_0000517* could probably be a sponge for *miR-326/miR-330-5p* in NSCLC cells.

### miR-326/miR-330-5p targeted MMP2 in NSCLC cells

**Fig.4:**
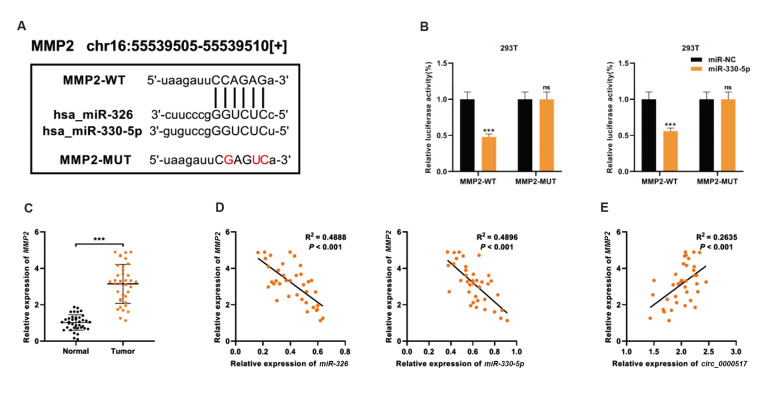
*MMP2* was a common target of *miR-326* and *miR-330-5p*. **A.** Bioinformatics analysis predicted that the sequence of *MMP2* 3ˊUTR matched the sequences of *miR-326/miR-330-5p*. MMP2-WT and MMP2-MUT luciferase reporter vectors were constructed. **B.** Dual-luciferase reporter assays demonstrated that *MMP2* was the direct target of *miR-326/miR-330-5p*. **C. **qRT-PCR was performed to examine *MMP2* expression in 37 paired NSCLC tissues and matched adjacent normal tissues. **D.** Pearson’s correlation analysis was utilized to evaluate the correlations between *MMP2* and *miR-326/miR-330-5p* expression levels in NSCLC tissues. **E.** Pearson’s correlation analysis was utilized to evaluate the correlation between *circ_0000517* expression and *MMP2* expression in NSCLC tissues. ***; P<0.001, ns; Not significant, NSCLC; Non-small cell lung cancer, and qRT-PCR; Quantitative real-time polymerase chain reaction.

Using StarBase online database, *MMP2* was predicted to be a common downstream target of *miR-326* and *miR-330-5p* (Tables S2, S3, See Supplementary Online Information at www.celljournal.org). The TCGA database showed that the overall survival of NSCLC patients with high *MMP2* expression was relatively shorter (Fig . S1, See Supplementary Online Information at www. celljournal.org). Additionally, wild-type MMP2 3ˊUTR (MMP2-WT) and mutant MMP2 3ˊUTR (MMP2-MUT) luciferase reporter vectors containing the *miR-326/miR-330-5p* binding site were constructed (Fig .4A). The data of the luciferase reporter gene assay showed that *miR-326/miR-330-5p* mimics remarkably weakened the luciferase activity of MMP2-WT reporter, but exerted no remarkable influence on the luciferase activity of MMP2- MUT reporter (Fig .4B). The data of qRT-PCR revealed that *MMP2* mRNA was overexpressed in NSCLC tissues (Fig .4C). Furthermore, *MMP2* expression in NSCLC tissues was negatively correlated with *miR-326/ miR-330-5p* expression and positively correlated with *circ_0000517* expression (Fig .4D, E). Therefore, it was hypothesized that *circ_0000517* positively regulated MMP2 expression by down-regulating *miR-326/miR-330-5p* in NSCLC cells.

### *Circ_0000517 *facilitates NSCLC cell growth and metastasis via the miR-326/miR-330-5p-MMP2 axis

**Fig.5:**
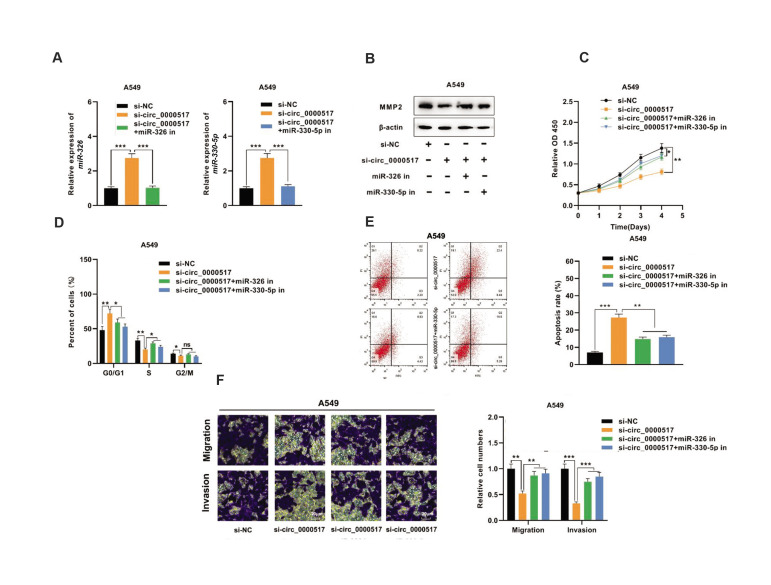
*Circ_0000517* facilitates NSCLC progression by acting on the miR-326/miR-330-5p-MMP2 axis. **A. **A549 cells with circ_0000517 knockdown were co-transfected with miR-326/miR-330-5p inhibitors or the negative control. qRT-PCR was employed to determine *miR-326/miR-330-5p* expression.** B.** Western blot was adopted to detect MMP2 protein expression in A549 cells co-transfected with circ_0000517 siRNA and miR-326/miR-330-5p inhibitors. **C.** CCK-8 assay was applied to detect the proliferation of A549 cells after their transfection. **D.** Flow cytometry was utilized to detect the cell cycle stage of A549 cells after the transfection. **E. **Flow cytometry was conducted to detect the apoptosis of A549 cells after their transfection. **F.** Transwell experiment was employed to detect the migration and invasion of A549 cells following transfection. *; P<0.05, **; P<0.01, ***; P<0.001, ns; Not significant, NSCLC; Non-small cell lung cancer, and qRT-PCR; Quantitative real-time polymerase chain reaction.

To elaborate on whether *circ_0000517* affected NSCLC progression through the circ_0000517-miR-326/miR-330-5p-MMP2 axis, miR-326/miR-330-5p inhibitors (or control) were transfected into A549 and H1299 cells along with *circ_0000517* knockdown (Fig .5A). Western blot results suggested that knocking down *circ_0000517* impeded MMP2 expression, whereas down-regulating *miR-326/miR-330-5p* reversed this effect (Fig .5B). Besides, functional compensation experiments were executed in A549 cells. CCK-8 experiments showed that the inhibition of *miR-326/miR-330-5p* diminished the suppressive effect of down-regulation of *circ_0000517* on A549 cell proliferation (Fig .5C). Flow cytometry analysis revealed that co-transfection with miR-326/miR-330-5p inhibitors reversed the effects of *circ_0000517* knockdown on cell cycle progression and apoptosis (Fig .5D, E). Furthermore, Transwell experiments revealed that co-transfection of miR-326/miR-330-5p inhibitors counteracted the effects of *circ_0000517* knockdown on migration and invasion of A549 cells (Fig .5F). In summary, *circ_0000517* exerted oncogenic effects in NSCLC by regulating miR-326/miR-330-5p-MMP2 axis.

## Discussion

NSCLC is one of the deadliest threats to human health (20). Despite the emergence of a variety of new treatment strategies for NSCLC, most patients still show poor prognosis due to metastasis and recurrence (21). In recent years, an increasing number of research demonstrates the importance of circRNAs in cancer biology (22, 23) and circRNAs have become a hotspot in cancer research. Aberrantly expressed circRNAs are reported to serve as biomarkers for the early diagnosis of several human cancers, such as gastric cancer, HCC, glioma, and prostate cancer (24, 25). Importantly, more and more studies reveal that circRNA is associated with NSCLC development. For instance, *circ_0001946* is down-regulated in NSCLC, and knocking it down enhances cancer cell proliferation, migration, and invasion, while restraining apoptosis (26). *Circ_0067934* expression, however, is up-regulated in NSCLC, and its overexpression is markedly linked to low survival, which can be an independent factor affecting the prognosis of NSCLC patients (27). Furthermore, *circ_0017247* is overexpressed in NSCLC, and knocking it down prevents cancer cell metastasis and epithelial-mesenchymal transition (28). In the current work, the analysis of GSE158695 revealed that *circ_0000517* was up-regulated in NSCLC tissue specimens. *Circ_0000517* is transcribed from the *RPPH1* gene on chromosome 14:20811404-20811492 (29). Reportedly, *circ_0000517* is remarkably up-regulated in HCC and is related to high tumor, nodes, and metastases (TNM) staging (13). We demonstrated that *circ_0000517* is also highly expressed in NSCLC tissues and cells in the present study. The upregulation of *circ_0000517* was closely associated with higher clinical stage, lymph node metastasis, and poor prognosis in NSCLC patients. Knockdown of *circ_0000517* blocked the proliferation, migration, and invasion of NSCLC cells, while enhancing apoptosis. These findings indicate that *circ_0000517* is an oncogenic factor in NSCLC.

Reportedly, circRNAs mainly work as miRNA sponges to adsorb miRNAs and modulate target genes’ expression, thereby exerting either pro- or anti-cancer effects (30). *Circ_0000517* is reported to modulate the expression levels of IGF1R and SMAD6 via sponging *miR-326* in HCC (29, 31). Furthermore, *circ_0000517* interacts with miR-1296-5p to increase *Txndc5* expression, facilitating the proliferation of HCC cells and repressing cell cycle arrest and apoptosis (32). In this work, *circ_0000517* was found to target and inhibit *miR-326* and *miR-330-5p* expression in NSCLC. Both *miR-326* and *miR-330-5p* are reported to be under-expressed in NSCLC; *miR-326* and *miR-330-5p* overexpression impedes the proliferation and invasion of NSCLC cells and suppresses tumor growth (33-35). In this work, compensation assays indicated that *miR-326/miR-330-5p* down-regulation partially counteracted the inhibitory effects of *circ_0000517* depletion on NSCLC cells. These findings suggest that *circ_0000517* works as a ceRNA to exert an oncogenic effect in NSCLC by modulating *miR-326* and *miR-330-5p* expression.

MMP2 is a matrix metalloproteinase that belongs to a large family of zinc-dependent endopeptidases (36). Mounting research demonstrates that aberrant MMP2 expression in diverse cancers is linked to tumor aggressiveness. For instance, by mediating MMP2 expression and activity in melanoma cells, long non-coding RNA (lncRNA) *GAS5* represses the invasion of cancer cells (37). *MiR-29b* inhibits gastric cancer tumor growth and cell migration through negatively regulating MMP2 (38). ROCk2 enhances HCC invasion and metastasis by modifying MMP2 ubiquitination and degradation (39). Importantly, high *MMP2* expression in NSCLC tissues indicates poor prognosis of the patients, and it is also a crucial effector of many oncogenic pathways to promote the malignant phenotypes of NSCLC cells (40). In this work, we reported that MMP2 was negatively regulated by *miR-326/miR-330-5p* and positively regulated by *circ_0000517*. Our work provides a new explanation regarding the mechanism of MMP2 dysregulation in NSCLC. 

## Conclusion

Taken together, this work demonstrates that *circ_0000517* is up-regulated in NSCLC tissues and cells. *Circ_0000517* knockdown impedes NSCLC cell proliferation and metastasis and thus, enhances apoptosis. Mechanistically, *circ_0000517* is implicated in NSCLC development by acting on the miR-326/miR-330-5p-MMP2 axis. Nonetheless, this work is limited by* in vitro* experiments, and remains to be confirmed by future *in vivo* experiments in animal models.

## Supplementary PDF


