# Supplementary material for: Circular RNA circ_0000517 Facilitates The Growth and Metastasis of Non-Small Cell Lung Cancer by Sponging miR-326/miR-330-5p
Source: Cell J. 2021 Oct 30;23(5):552–61. doi: 10.22074/cellj.2021.7913 (PMC8588814; doi:10.22074/cellj.2021.7913)
Supplement: Supplementary file 1 [file Cell-J-23-552-s01.pdf]

Supplementary Information for

Circular RNA *circ_0000517* Facilitates The Growth and Metastasis of Non-Small Cell Lung Cancer by Sponging *miR-326/miR-330-5p*

Qiyan Tan, B.Sc.#, Changyu Liu, B.Sc.#, Ying Shen, B.Sc., Tao Huang, M.Sc.*

Department of Laboratory, Hainan People's Hospital, Haikou, Hainan, China

#These authors contributed equally to this Work.

***Corresponding Address: Department of Laboratory, Hainan People's Hospital, Haikou, Hainan, China**
Email: scy610463@163.com

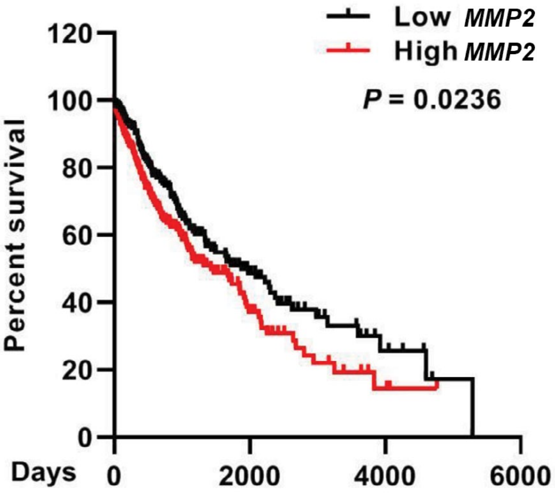

Fig.S1: Kaplan-Meier survival curve was used to analyze the relationship between the expression of *MMP2* and the overall survival of NSCLC patients (in TCGA database).

Table S1: Primer sequence used in this study

Gene	Primer sequence (5´-3´)
circ_0000517	F: GGGAGGTGAGTTCCCAGAG
	R: CAGGGAGAGCCCTGTTAGG
RPPH1	F: GTCACTCCACTCCCATGTCC
	R: CAGCCATTGAACTCACTTCG
MMP2	F: CTGGGAGCATGGCGATGGATA
	R: GGAAGCGGAATGGAAACTTG
miR-326	F: CATCTGTCTGTTGGGCTGGA
	R: AGGAAGGGCCCAGAGGCG
miR-330-5p	F: TCTCTGGGCCTGTGTCTTAG
	R: CAGTGCGTGTCGTGGAGT
β-actin	F: GGAGCGAGATCCCTCCAAAAT
	R: GGCTGTTGTCATACTTCTCATGG
U6	F: ATTGGAACGATACAGAGAAGATT
	R: GGAACGCTTCACGAATTTG
